# Core clock gene *Bmal1* deprivation impairs steroidogenesis in mice luteinized follicle cells

**DOI:** 10.1530/REP-20-0340

**Published:** 2020-09-17

**Authors:** Yizi Wang, Minghui Chen, Jian Xu, Xinyan Liu, Yuwei Duan, Canquan Zhou, Yanwen Xu

**Affiliations:** 1 Reproductive Medicine Center, The First Affiliated Hospital of Sun Yat-sen University, Guangdong, Guangzhou, China; 2 Reproductive Medicine Center, Guangzhou Women and Children’s Medical Center, Guangzhou Medical University, Guangzhou, China; 3 Guangdong Provincial Key Laboratory of Reproductive Medicine, First Affiliated Hospital of Sun Yat-sen University, Guangzhou, China

## Abstract

Luteinization is the event of corpus luteum formation, a way of follicle cells transformation and a process of steroidogenesis alteration. As the core clock gene, *Bmal1* was involved in the regulation of ovulation process and luteal function afterwards. Till now, the underlying roles of luteinization played by *Bmal1* remain unknown. To explore the unique role of *Bmal1* in luteal steroidogenesis and its underlying pathway, we investigated the luteal hormone synthesis profile in *Bmal1* knockout female mice. We found that luteal hormone synthesis was notably impaired, and phosphorylation of PI3K/NfκB pathway was significantly activated. Then, the results were verified in in vitro cultured cells, including isolated *Bmal1* interference granulosa cells (GCs) and theca cells (TCs), respectively. Hormones levels of supernatant culture media and mRNA expressions of steroidogenesis-associated genes (*star*, *Hsd3β2*, *cyp19a1* in GCs, *Lhcgr*, *star*, *Hsd3β2*, *cyp17a1* in TCs) were mutually decreased, while the phosphorylation of PI3K/NfκB was promoted during in vitro luteinization. After PI3K specific-inhibitor LY294002 intervention, mRNA expressions of* Lhcgr* and *Hsd3β2* were partially rescued in *Bmal1* interference TCs, together with significantly increased androstenedione and T synthesis. Further exploration in TCs demonstrated BMAL1 interacted directly but negatively with NfκB p65 (RelA), a subunit which was supposed as a mediator in *Bmal1*-governed PI3K signaling regulation. Taken together, we verified the novel role of *Bmal1* in luteal steroidogenesis, achieving by negative interplay with RelA-mediated PI3K/NfκB pathway.

## Introduction

Brain and muscle Arnt-like 1 (*Bmal1*) is the core component of the internal circadian clock system, which has been proved to be indispensable to maintain the mammalian circadian rhythms (Huang *et al.* 2012, Sellix 2015). *Bmal1* deprivation could bring about endogenous or entrainable oscillation alteration (Boden *et al.* 2010). Up to 10% of transcriptome in human was expressed in circadian manner (Panda *et al.* 2002). However, rhythmicity of subordinate clocks was not completely governed by single master pacemaker but showed tissue-specific characteristics (McDearmon *et al.* 2006, Zhang *et al.* 2014). Recently, extensive non-circadian regulation patterns of *Bmal1* were confirmed in many peripheral biological processes (Alvarez *et al.* 2003). For example, germline *Bmal1* loss results in an acceleration of aging but adult-life inducible knockout ones do not have such gross effects, while both are deficient in the circadian cock (Yang *et al.* 2016).

In ovary, luteinization is a transient process orchestrated by endocrine, paracrine and autocrine signals in a timely manner after ovulation. Both granulosa and theca cells in the ovulated follicle undergo luteinization to form corpus luteum, which are responsible for the synthesis of progesterone and estrogen required for maintaining early pregnancy before the placenta establishment. Evidences from previous studies have implicated that *Bmal1* was involved in the regulation of ovulation process and luteal function afterwards (Boden *et al.* 2010, Pan *et al.* 2020, Sen & Hoffmann 2020). Epidemiologically, shift on sleep-wake schedules might lead to spotting or other abnormalities in luteal phase (Shibui *et al.* 2000, Wang *et al.* 2016). Physiologically, verified by immunohistochemistry, BMAL1 expression significantly increased during corpus luteum formation (Wiggins & Legge 2016). Female mice with* Bmal1* knockout were inspected with impaired steroidogenesis, corpus luteum defect, and consequentially increased risks of implantation failure (Ratajczak *et al.* 2009, Boden *et al.* 2010, Sellix 2015). Furthermore, serum progesterone concentrations were at a comparative lower levels at the 3.5 day of gestation in Bmal1−/− pregnant mice (Ratajczak *et al.* 2009), a primary pregnancy stage when endogenous hormones were maintained mainly by corpus luteum prior to placenta formation.

The defects in ovulation and luteum function in Bmal1−/− mice were mainly considered to be due to the abolishment of circadian regulation on HPO (*hypothalamic–pituitary–ovarian*) axis. However, peripheral roles of *Bmal1* in ovary could not be ignored. In the mouse model with *Bmal1* specifically abolished in ovaries generated in the study of *Liu* et al., ovarian-targeted *Bmal1* deprivation evoked embryonic implantation impairment and simultaneously compromised serum progesterone concentrations (Liu *et al.* 2014). Subsequently, ovarian cell type-specific *Bmal1* knockout mice were generated by Mereness et al. Analogic phenotype as premature primordial follicle aggregation observed in Bmal1−/− mice was merely detected in mice with targeted deletion of the *Bmal1* locus in theca cells (TCs) (TCKO), but not in those females with granulosa cells (GCs) *Bmal1* knockout (GCKO) (Mereness *et al.* 2016). When concerning luteinization, speculations were raised whether regulations governed by *Bmal1* were achieved mainly through ovarian TCs other than GCs, although the majority studies to date had tended to emphasis GCs’ roles rather than TCs’.

Till now, molecular mechanisms of *Bmal1* in ovary underlying early luteinization after ovulation still remain elusive and definitely deserve further exploration. By sequence comparison to classic circadian clock-controlled *cir*-regulatory elements (E-box, D-box and RORE) (Takahashi 2017), several steroidogenesis associated genes expressed in both GCs and TCs (*Star*, *Lhcgr*, *Hsd3β2*, and *Ptgs2*), exclusively in GCs (*Fshr*, *Cyp19a1*), or exclusively in TCs (*Cyp17a1*) were detected in presence with one or more aforementioned elements in their promoters. In consideration of the aforementioned evidences, the implicated hypothesis about whether *Bmal1* affected luteal hormone synthesis through entraining steroidogenic-associated genes with clock-controlled promoters directly or via other cellular mechanisms still need to be unraveled.

Given GCs and TCs’ critical involvement in steroidogenesis and their cell-specific characteristics during luteinization, it is imperative to further explore the roles of *Bmal1* in these two kinds of cells separately. Therefore, the main purpose of the present study was to investigate the assigned roles of *Bmal1* in cultured GCs and TCs, respectively, during luteinization, and to explore relevant cellular mechanism using both *Bmal1* knockout mouse model and *Bmal1*-knockdown cultured cells in vitro.

## Materials and Methods

### Animals

Heterozygous* Bmal1* knockout mice on C57BL/6J background was purchased from Nanjing Biomedical Research Institute of Nanjing University. Age-matched WT C57BL/6J mice were purchased from the Guangdong Province Laboratory Animal Center. Female *Bmal1−/*− offspring were developed by heterozygous pairs mating, whose genotype were determined as previously described (Bunger *et al.* 2000). The feeding conditions and Zeitgeber time definition were described in our published paper (Xu *et al.* 2016), as from 06:00 h (Zeitgeber time 0, ZT 0) light: 18:00 h darkness cycle (ZT 12). All experimental protocols, including animals were approved by the Ethical Committee of the First Affiliated Hospital of Sun Yat-Sen University.

### Serum hormone measurements

The estrous of 8-week-old female mice were synchronized as previously described (Park *et al.* 2014). One week later, females received an intraperitoneal injection (i.p.) of 15IU pregnant mare serum gonadotrophin (PMSG; Ningbo Second Hormone Factory, Ningbo, China) at 08:00 h (ZT 2) followed by 10IU human chorionic gonadotropin (HCG, sigma) after 48 h. Twenty-four, 36, 48 h (functional stage of luteal phase) and 72, 96 h (regression stage) later after PMSG/HCG injection (Park *et al.* 2014), whole blood samples were over time collected by cardiac puncture just after sacrificed by cervical dislocation. Before centrifugation, blood was allowed to coagulate at room temperature for 1 h. Serum was separated according to manufacture protocol and stored at −80°C until assayed. Estradiol and progesterone levels were measured analyzed by RIA using commercial iodine [^125^I] RIA Kits (Beijing North Biotechnology Research Institute). The sensitivity of the progesterone and estradiol RIA assays was 20 ng/mL. The intra-assay error and inter-assay error were <10 and <15%, respectively.

### Isolation and identification of granulosa cells and theca interstitial cells

Four- to 5-week-old SPF WT female mice at proestrus stage were super-ovulated by an intraperitoneal injection (i.p.) of 15IU pregnant mare serum gonadotrophin (PMSG; Ningbo Second Hormone Factory, Ningbo, China) at 08:00 h (ZT 2). Mice were humanely sacrificed by cervical dislocation 48 h after injection.

By puncturing follicles from the isolated ovaries with a 26 gauge needle immediately, granulosa cells were liberated and collected into precooled DMEM-F12 media. Residual tissue was reserved for TCs collection. GCs suspensions were filtered through 40 μm nylon filter and then centrifuged at 250 ***g*** for 5 min. Sediments were collected and centrifugation procedure was repeated twice. Supernatant was discarded and the cells seeded into a 24-well culture plated (2x10^5^ cells/well). Every 2–3 days, half of the cultured DMEM/F-12 (1:1) media (containing 100 U/mL penicillin, 100 U/mL streptomycin, 15% fetal bovine serum and 10 ng/mL EGF) was replaced with fresh one. TCs isolation was performed according to the method previously described (Tian *et al.* 2015) with slight modification as follows: 4 mL of 33% Percoll was layered on top of the 45% Percoll solution, on top of which the dispersed cells were gently layered, and tubes were centrifuged at 400 ***g*** for 15 min. Then by aspiration inside the 33% Percoll layer followed by centrifugation at 400 ***g*** for 5 min, the sediments were collected. The final pellet of TCs was resuspended in the reported culture media volume after washing twice with McCoy’s 5a medium.

Isolated cells were counted with a hemacytometer and a trypan blue staining was applied to test its viability. FSHR (follicle-stimulating hormone receptor) and CYP17A1 antibody, as specific marker enzyme expressed in GCs and TCs, respectively, was utilized to verify the purity and cell types according to a previous report by immunofluorescence staining (Chen *et al.* 2013*a*). Briefly, after 4% paraformaldehyde fixation and 0.1% Triton X-100 penetrating cell membrane, cells were incubated overnight at 4°C with rabbit anti-mouse FSHR antibody (1:200 dilution, Novus) and rabbit anti-mouse CYP17A1 (1:200 dilution, Santa Cruz). After washing in PBS, the cells were incubated for 1 h at 37°C with a secondary biotinylated donkey anti-rabbit IgG antibody (dilution 1:300, Santa Cruz). The cells were then washed in PBS and incubated for 10 min at 37°C with DAPI dye liquor. The staining of FSHR was recorded with a laser confocal microscope (Olympus CKX41). A positive staining was evaluated by a green fluorescence for CYP17A1 (1:200 dilution, Santa Cruz), and blue fluorescence represented FSHR (1:200 dilution, Novus).

### MTS

Ovarian cell viability was evaluated based on MTS method by applying MTS assay kit (Abcam, ab197017) according to the manufacturer's instructions. In brief, 48-h post*-Bmal1* siRNA transfection (as zero point at horizontal axis), 20 μL/well of the MTS was added to cultured TCs and GCs (5 × 10^4^ cells/well in 180 μL fresh medium in 96-well plates) for an additional 4 h of incubation under 37°C, respectively, at 24-, 48- and 72-h point in time. Untransfected cells were served as the control. Absorbance at OD = 490 nm was measured using microplate reader (Daojin UV-2450), and results were expressed as a percentage of the untreated control.

### siRNA transfection

Three systemic siRNA targeting *Bmal1* with fluorescence-FITC labeling for mice were delivered into primary theca cells using Lipofectamine 3000 transfection kit (Invitrogen Corp) 1 day after cell plating when the cell density reached 60%. The planted density was 5 × 10^5^ at each well in six-well plant with 5 µL/well FITC siRNA-Lipofectamine 3000 dilution medium without serum each well to a final concentration of 50 nM. Meanwhile, the non-silencing RNA was transferred in the same condition with merely lipo complex (Mock) and PBS (NC) as controls (all of the siRNA were synthesized by RuiBo Biotechnology Company, Guangzhou). The process was lucifugal. Candidate sequences of RNA oligos were listed in Table 1. Among them, the first one with the highest transfection effect was finally chosen for the subsequent experiment. Transfection was processed according to the manufacturer’s instructions. Visible green FITC-siRNA fluorescence and quantitative *Bmal1* mRNA extraction were prepared to test the efficacy of siRNA 48 hours later. Cells were harvested at the indicated time points and processed for further analysis. After transfection, cells were resuspended in regular culture medium and plated. Proteins of target pathway were analyzed by Western blot analysis using specific antibodies. Nf-κBp65 siRNA was a commercial product (CST, 6337S).

**Table 1:** siRNA sequences targeting *Bmal1* mRNA.

Gene	siRNA target sequence (5’–3’)
*Bmal1*	
1	CCAAGGAAGTTGAATACAT
2	GCTCTTTCTTCTGTAGAAT
3	GCAAACTACAAGCCAACAT

### Measuring hormone synthesis and sensitivity to LH stimulation in vitro

Culture supernatants from GCs or TCs monolayers were taken from individual wells to detect hormone levels 48 h after siRNA transfection with additional 12-h LH co-culture. After centrifugation at 2500 ***g*** at 4°C for 15 min, the supernatant was stored at −80°C for hormone measurement. The concentrations of Estradiol (E_2_; KGE014, R&D Systems), testosterone (T, RnD, KGE010), progesterone (P_4_, EA, Merck-Millipore, STTHMAG-21K-02) and androstenedione (AND, LSBio, ELISAKit-LS-F39181) were determined by ELISA using kits according to the manufacturer's protocols, respectively. A reported physiological concentration (2.5 ng/mL) of luteinizing hormone (LH) was added for 12 h to induce and maintain hormone secretion. Intra- and inter-assay precisions as described by coefficients of variations were T, ≤3.1% and ≤6.3%; AND, <8% and 10%. The detection limits of E_2_, T, P_4_ and AND were 12.3–3000 pg/mL, 0–10 ng/mL, 0.156–10 ng/mL, and 0.156–10 ng/mL, respectively.

### LY294002 treatment

Post-siRNA transfection to *Bmal1* impairment, LY294002 (Sigma, L9908-LMG) as a PI3K inhibitor, was (200 µM) co-cultured with cells for 2 h at 37°C to suppress the phosphorylation of PI3K pathway.

### Quantitative real-time PCR analysis

GCs and TCs were harvested and RNA was extracted using Trizol reagents according to manufacture instructions (Invitrogen). Quality and concentration of total RNA were checked using NanoDrop (Thermo Fisher Scientific). Samples were sequentially treated with RNase-free DNase I (TaKaRa) and Superscript II reverse transcriptase (Invitrogen) to remove contaminating genomic DNA and reverse transcribed into cDNA. qRT-PCR was performed in triplicate according to protocols (Xu *et al.* 2015). The sequences of the primers for core circadian genes (*Per1*, *Per2*, *Cry1*, *Cry2*, *Clock*, *Bmal1*) and hormone synthesis related genes (*Fshr*, *Lhcgr*, *Star*, *Cyp11a1*, *Hsd3β2*, *Cyp19a1* and *Cyp17a1*) were listed in Table 2. Transcript levels were normalized with that of the housekeeping gene *Gapdh*.

**Table 2:** Primer sequences for mice targeted steroidogenesis-associated genes and circadian clock genes.

Gene	Primers sequence (5’–3’)	
	Forward	Reverse
*Bmal1*	CCGTGGACCAAGGAAGTAGA	CTGTGAGCTGTGGGAAGGTT
*Star*	GGGTGGATGGGTCAAGTTC	AGCACTTCGTCCCCGTTC
*Cyp11a1*	GTCCCACTCCTCAAAGCCAG	GAAGCACCAGGTCGTTCACAAT
*Hsd3β2*	GCCCCTACTGTACTGGCTTG	TCCCGATCCACTCTGAGGTT
*Cyp17a1*	GCCCAAGTCAAAGACACCTAAT	GCCCAAGTCAAAGACACCTAAT
*Cyp19a1*	ACCTGGAGTAGGAGCCTTTACCTGC	CAGGTCCTGTTCAGCGGTTGGT
*Fshr*	TGAGTCTGGCTATGCGTGTCTA	CACCTCATAACAGCCAAAC
*Lhcgr*	GAGACGCTTTATTCTGCCATCT	CAGGGATTGAAAGCATCTGG
*NFκB*	TGATCCACATGGAATCGAGA	CAGGAAGGGATATGGAAGCA
*Clock*	GGAGTCTCCAACACCCACAG	GGCACGTGAAAGAAAAGCAC
*Per1*	AGATCAACTGCCTGGACAGC	AGATCAACTGCCTGGACAGC
*Per2*	TGGCCTCCATCTTTCACTGT	CAACACTGACACGGCAGAAA
*Cry1*	CCATCCGCTGCGTCTATATCC	GAAGCAAAAATCGCCACCTGT
*Cry2*	AATTCCTTACTGGCCAGCCC	TTCTCGCCACAGGAGTTGTC
*Gapdh*	TGTAGACCATGTAGTTGAGGTCA	AGGTCGGTGTGAACGGATTTG

### Western blot analysis

After centrifugation (10,000 ***g***, 15 min at 4°C), the supernatants were collected for protein analysis. The protein concentration was determined by Bradford protein assay. Western blot analysis was performed as previously described. Briefly, 50 µg proteins from each sample were loaded onto an SDS polyacrylamide gel for electrophoresis and subsequently transferred to polyvinylidene fluoride membranes. The membranes were blocked with 5% nonfat dry milk in PBS for an hour and incubated with a primary antibody against BMAL1 (1:200 dilution), AKT (1:1000 dilution), p-AKT (Thr 308) (1:2000 dilution), mTORC1 (1:1000 dilution), p-mTORC1 (Ser2481) (1:2000 dilution), S6K1 (1:1000 dilution), p70-S6K1 (Thr389) (1:2000 dilution), NfκBp65 (dilution 1:250 dilution), p-NfκB (Ser536) (1:250 dilution) overnight at 4°C (BMAL1, mTORC1, p-mTORC1, NfκBp65 and p-NfκB were purchased from Abcam; AKT, p-AKT, S6K1S and p70-S6K1 were purchase from CST Danvers, USA). Samples were then incubated for 1 h at room temperature with horseradish peroxidase-conjugated secondary antibodies (Santa Cruz, USA) (1:5000 dilution). Band intensities were visualized with chemiluminescence reagent (Millipore Corp.) by the BioMax film (Kodak) and then analyzed with Gel-Pro analyzer 4.0 software. β-Actin (Santa Cruz, USA) protein was utilized as a loading control.

### ***Bmal1*** overexpression in theca cells

A lentivirus overexpression vector with the *Bmal1* and enhanced green fluorescent protein gene (eGFP) insert (LV5-eGFP-*Bmal1*, GenePharma, Shanghai, China) was constructed successfully and packaged into high-titer lentiviruses. The primary mice TCs were transfected with recombinant virosome or empty vector when the cell density was approximately 50%. Expression of eGFP protein in each group was observed under an inverted fluorescence microscope at 5 days after transfection. The infection efficiency was confirmed by qRT-PCR.

### Immunoprecipitation (IP)

All steps were performed at 4°C or on ice for immunoprecipitation after overexpression of BMAL1 in the primary theca cells.

Cells were washed twice with ice-cold PBS after gentle aspiration of culture media and lysed with lysis buffer containing protease inhibitor (Roche) for 10 min. Whole-cell protein lysates were next centrifuged at 16,000 ***g*** for 10 min and normalized against a standard curve of BSA by Bradford assay. One milligram of total protein was washed with DynaBeads Protein G magnetic beads (Invitrogen) for 15 min. Supernatants were transferred and incubated with 3 µg of mice anti-BMAL1 antibody (Abcam)/µg of total protein at 4°C overnight with constant agitation, or mice IgG (Abcam) for negative control. A total of 30 µL of pre-washed magnetic beads were added to per lysates for half an hour with constant agitation to capture the BMAL1-combined protein complex. Immobilized fractions were washed at least three times with ice-cold lysis buffer, including protease and phosphatase inhibitors. All lysis buffer was instantly centrifuged at 16,000 ***g*** for 5  s. The supernatant was collected, then 35 µL of 2x SDS sample buffer was added, followed by boiling for 5 min at 95°C.

The complex was separated on an SDS-PAGE gel, and transferred to PVDF membrane. Anti-BMAL1 input with use of anti-BMAL1 as the probe was showed in Lane 1 (BMAL1 + cell samples). The enrichment in Panel 1 was sampling alone, and residual proteins in Panel 2 and 3 were jointly sampled. Lane 2 was performed with use of the anti-IgG antibody (Lane 2, IgG + cell samples) and Western blot of mere antibody IgG (Lane 3, mere IgG). Content in Panel 4 (of Lane 2) was sampled alone, and Panel 5 and 6 were sampled together, while the residuals in Lane 2 were sampled as whole. Proteins appearing in both Lane 2 and Lane 3 were identified as non-specific. After rolling out the non-specific ones from those proteins appeared at Lane 1, the specific proteins that direct interacted with BMAL1 were obtained. Abundance of membranes from BMAL1 immunoprecipitates were determined and categorized by mass spectrometric analysis (Q Exactive mass spectrometer, Thermo Fisher).

### Statistical analysis

Results from Western blot were quantified with the ImageJ software. Statistical analysis was performed by applying GraphPad Prism (version 5.0) and Student’s *t*-test. For comparison between two groups, a one-way ANOVA was performed followed by Dunnett’s correction. Values of *P* < 0.05 were considered significant and indicated by asterisks in the figures.

## Results

### Deprivation of ***Bmal1*** impaired luteal function in vivo

**Figure 1:**
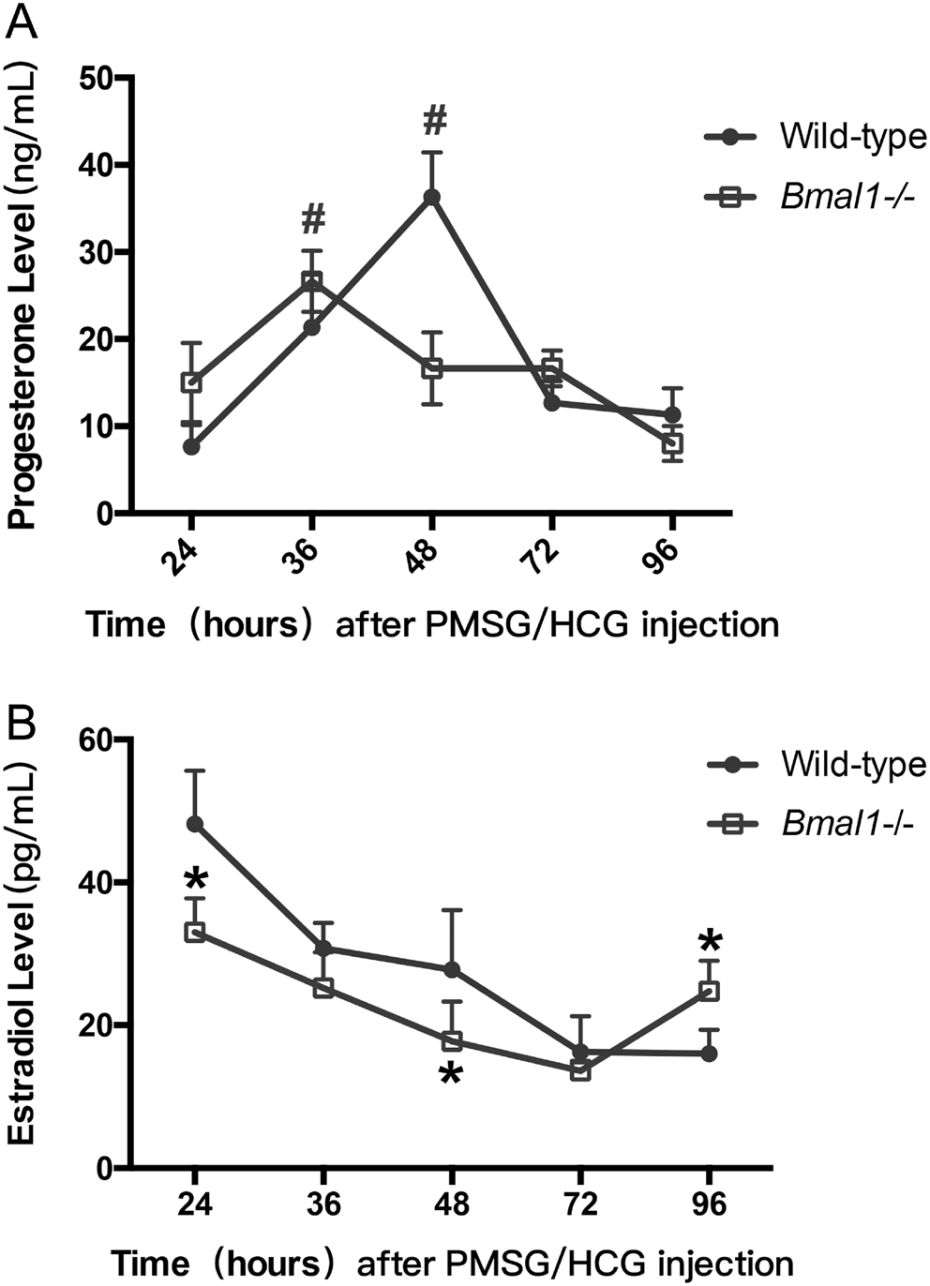
Luteal profiles of serum hormone levels through out 4 days post-PMSG/HCG injection. Hormones were measured by ELISA kits. Each value represents the mean ± s.d. (A) Progesterone levels. #Peak value comparison between Bmal1−/− and WT female mice with *P*<0.05 or less. (B) Estradiol levels. *Comparison between Bmal1−/− and WT at corresponding labelled time point with *P <* 0.05 or less. *n* = 3 females at 8 weeks old per time point in each group.

We first investigated the change of progesterone levels at different time points post-PMSG/HCG injection in *Bmal1* knockout mice (Fig. 1A). Serum progesterone level peak was shown at 36 h following PMSG/HCG treatment in *Bmal1−/*− females, evidently advanced to the WT females which showed the progesterone peak 12 h later. The peak progesterone level was statistically reduced to 18.4 ± 5.6 ng/mL of *Bmal1* knockout females as compared to 36.2 ± 7.7 ng/mL of WT ones (*P* < 0.01). Serum estradiol level declined progressively overtime after luteinization within 72 h in the two genotypes (Fig. 1B). The estradiol concentrations were 37.3 ± 8.0 and 18.9 ± 6.1 pg/mL of *Bmal1−/*− mice at 24, 48 h, respectively, significantly lower than 56.1 ± 10.3 and 30.7 ± 11.0 pg/mL of WT at the same time points (*P* = 0.01, *P* = 0.03). Thereafter, serum E_2_ rose in advance in *Bmal1−/−* ones, reaching to 28.4 ± 4.4 pg/mL at 96 h, while the E_2_ remained at a lower level of 17.2 ± 5.7 pg/mL in WT (*P* = 0.03).

### *Bmal1* knockdown promoted *in vitro* follicle cell proliferation

**Figure 2 fig2:**
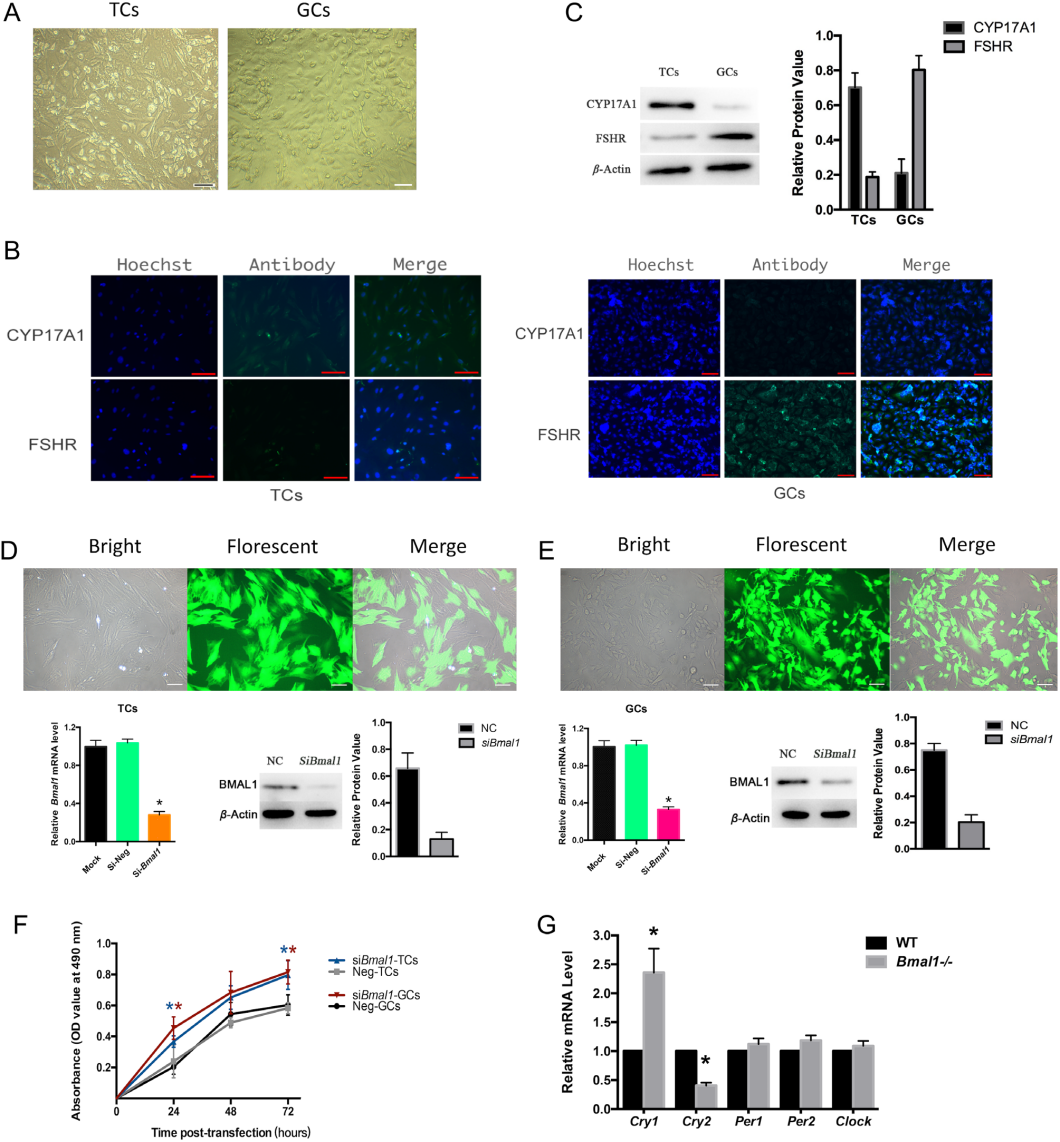
Follicle cells proliferation and transcriptions of other core circadian genes post-siRNA-Bmal1 transfection. (A) Cells morphology. Fusiform-aligned TCs and cobblestone-like GCs (200x, Scale Bar = 100 μm). (B) Immunofluorescence staining of CYP17A1 and FSHR observed under fluorescence microscope (400x, Scale Bar = 50 μm). Most isolated TCs remained CYP17A1 positive and FSHR negative, while immunofluorescence staining of CYP17A1 and FHSR showed opposite in GCs. (C) Relative protein levels of CYP17A1 and FSHR by Western blot (left) and corresponding band intensities (right). (D) and (E) Transfection efficiency of TCs (D) and GCs (E). Transfected cells were signaled by green fluorescence (upper) and knockdown efficiency in isolated cells (below) was ascertained by qRT-PCR analysis of Bmal1 mRNA. Both Mock (nonsiliencing) and si-Neg (empty vector transfected) were controls. *P < 0.05 vs the si-Neg group. (F) Growth curve according to OD value after siRNA-Bmal1 transfection detected by MTS assays. Bmal1 interference accelerated both isolated GCs and TCs growth significantly. (G) mRNA expression of core circadian genes (Cry1, Cry2, Per1, Per2, clock) in mice ovaries quantified by qRT-PCRT, WT mice. Bmal1−/−, mice genotyped with whole bmal1 knockout. *P < 0.05 or less. Data are presented mean ± s.d. of three independent determinations. *P < 0.05 or less vs the Neg (control) group. TCs, theca cell. GCs, granulosa cells.

TCs exhibited fibroblast-like, long fusiform or anomalistic triangular shapes, while GCs looked like cobblestones with polygonal or cuboidal shapes (Fig. 2A). Cells were further identified by Cytochrome P450, family 17, subfamily A, polypeptide 1 (CYP17A1), known to be specifically expressed in TCs, and the GCs-specific marker Follicle-Stimulating Hormone Receptor (FSHR). As shown in Fig. 2B,2 the majority isolated TCs remained CYP17A1 positive and FSHR negative, while GCs showed the opposite results. Additionally, total protein from the isolated TCs and GCs were extracted, respectively, for Western blotting of CYP17A1 and FSHR. As shown in Fig. 2C,2 FSHR was expressed at very low levels in the TCs, while CYP17A1 was expressed at high levels. Conversely, FHSR, but not CYP17Aa was expressed abundantly in the GCs.

*Bmal1* transcription rates were decreased to 27.34 ± 5.92% in TCs and 32.21 ± 4.40% in GCs, respectively, after siRNA transfection when compared to the controls by qRT-PCR (*P* < 0.01) (Fig. 2B). The transfection efficiency was confirmed by Western blot (Fig. 2D for TCs and Fig. 2E for GCs).

MTS assay was conducted to evaluate cells proliferation 72 h post-transfection. Growth curve was drawn according to OD value. As presented in Fig. 2F,2 cell growth of both TCs and GCs significantly accelerated after *Bmal1* siRNA transfection, as compared to the controls.

Considering feedback loops and compensated interactions between *Bmal1* and other clock genes, transcriptional levels of core circadian genes such as *Cry1*, *Cry2*, *Per1*, *Per2*, and *Clock* were tested in the ovaries of *Bmal1*^−/−^ mice. As shown, the mRNA expression of *Cry2* was significantly decreased, while *Cry1* was significantly increased in ovaries from *Bmal1* knockout mice. However, transcriptional expressions of *clock*, *Per1* and* Per2* were unaffected (Fig. 2G).

### Suppression of ***Bmal1*** impaired secretion of steroid hormones

To further investigate the role of *Bmal1* on luteal hormone secretion, we measured steroid hormone concentrations in the culture supernatants of cells subjected to LH stimulation for 12 h. Cells transfected with empty vector consisted of the sham control group.

**Figure 3 fig3:**
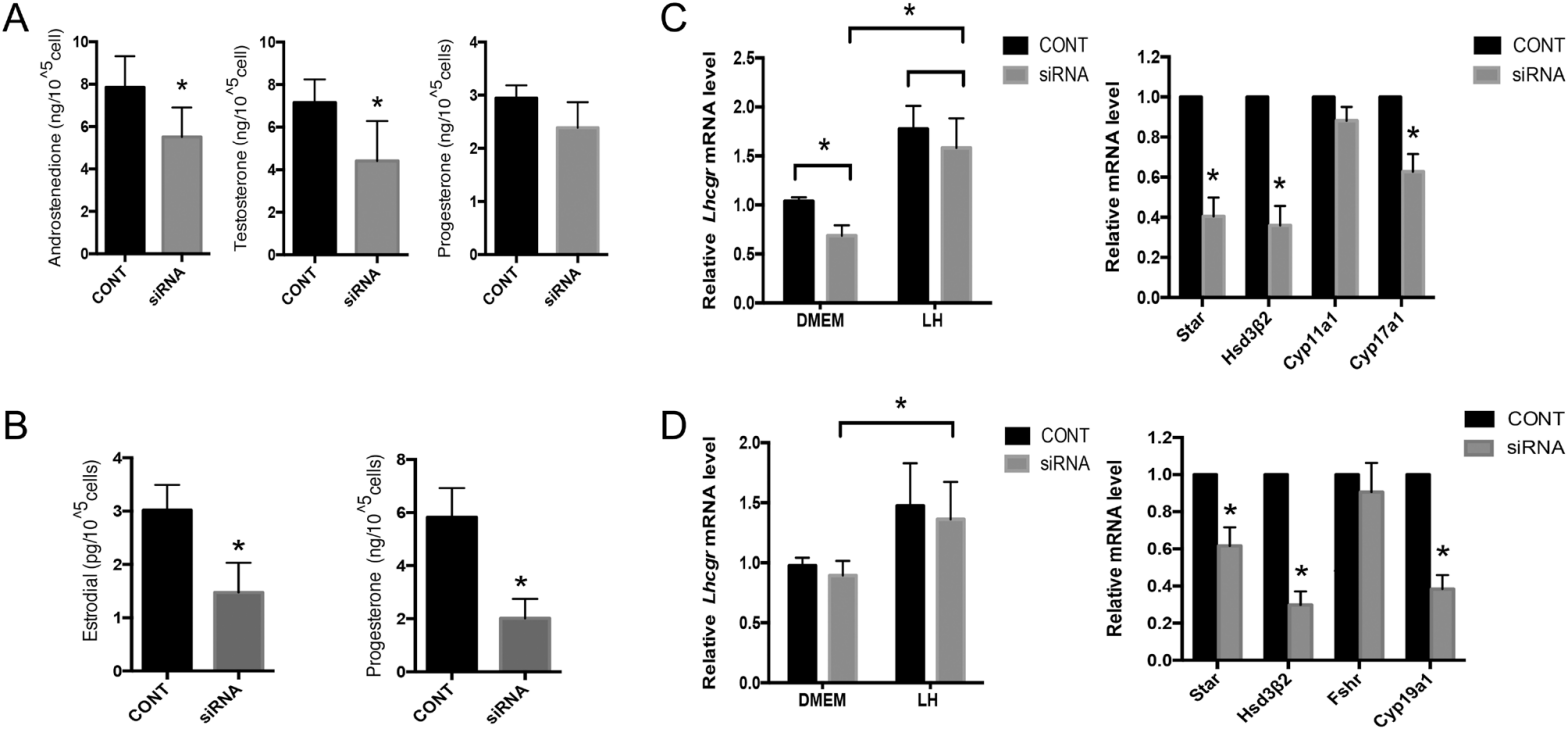
*Bmal1* downregulation decreased luteal hormones synthesis and impaired mRNA transcriptions of steroidogenesis-associated genes in lutenized follicle cells. (A) Androgen and progesterone concentrations in TCs’ supernatants. (B) Progesterone and estradiol levels in GCs’ supernatants. *Bmal1* interference deteriorated P_4_ and E_2_ production. (C) Left, TCs’ *Lhcgr* mRNA levels. Right, mRNA transcriptions of other targeted steroidogenesis-associated genes in TCs. (D) Left, GCs’ *Lhcgr* mRNA levels. Right, mRNA transcriptions of other targeted steroidogenesis-associated genes in GCs. The relative mRNA expression was normalized to *Gapdh* and expressed as relative to CONT. siRNA, group of cells transfected with siRNA against *Bmal1*. CONT, cells transfected with siRNA with empty vector as controls. LH, cell cultured with LH supply additionally for 12 h. DMEM, cells cultured with regular culture media containing DMEM. mRNA levels of target genes in cells were quantified by qRT-PCR using their specific primers. Data are presented mean ± s.d. of five independent determinations. *P < 0.05 or less vs the control group.

In TCs, both AND and T concentrations were significantly lower in the *Bmal1*-siRNA group (AND 5.51 ± 1.39 ng/10^5^ cell, T 4.41 ± 1.87 ng/10^5^ cell) than those in the controls (AND 7.85 ± 1.46 ng/10^5^ cell, T 7.15 ± 1.10 ng/10^5^ cell) (*P* = 0.006 and 0.033, respectively). Meanwhile, P_4_ levels were presented with down trend post-transfection (Fig. 3A). As presented in Fig. 3B,3 suppression of *Bmal1* impaired E_2_ and P_4_ synthesis in GCs (E_2_: 1.48±0.56 vs 3.02±0.47 pg/10^5^ cell, *P* = 0.009; P_4_: 5.82±1.01 vs 2.02±0.73 ng/10^5^ cell, *P* = 0.003).

A subset of genes related to steroidogenesis was further investigated after *Bmal1* siRNA transfection. In the *Bmal1*-siRNA group, the transcriptional level of *Lhcg*r in TCs significantly decreased (Fig. 3C, left), while no change was detected in GCs (Fig. 3D, left). However, the differences of *Lhcgr* levels in TCs between *Bmal1*-siRNA group and the control were vanished after LH stimulation, which raised comparability within groups (Fig. 3C, left). For other steroidogenesis-associated genes, in TCs, the mRNA levels of *Star*, *Hsd3β2*, and *Cyp17a1* in the *Bmal1*-siRNA group were significantly lower than control group after LH stimulation, except *Cyp11a1* (Fig. 3C, right). As in GCs, the mRNA levels of *Star*, *Hsd3β2*, and *Cyp19a1*, except FSHR, in the transfected group were significantly lower than the control (Fig. 3D, right). The reduced transcriptional levels of *Star*, *Cyp19a1*, *Hsd3β2*, and *Cyp17a1* were in accordance with decreased secretion of E_2_ and down trend of P_4_.

### ***Bmal1*** interference activated phosphorylation of PI3K/NfκB pathway

**Figure 4:**
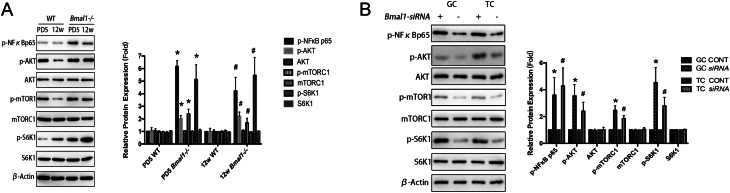
*Bmal1* loss activated phosphorylation of NFκB/PI3K pathway. (A) Ovarian protein levels and quantitation assessment of *Bmal1*-knockout mice by Western blot. Left, a typical Western blot. Right, bands were densitometrically qualified and the intensities shown were normalized to β-Actin, relatively to WT control. PD 5, post-natal 5 days. *Bmal1−/*−, mice genotyped with whole *bmal1* knockout. *P < 0.05 or less vs PD5 WT; #*P* < 0.05 or less vs 12 week WT. Data are presented mean ± s.d. of three independent experiments. (B) Protein levels and quantitation assessment of *Bmal1*-knockdown GCs and TCs with siRNA transfection. Left, a typical Western blot. Right, bands were densitometrically qualified and the intensities shown were normalized to β-actin, relatively to CONT of corresponding cell types. GC, granulosa cell. TC, theca cell. siRNA, group of cells transfected with siRNA knockdown *Bmal1* transcriptions. CONT, cells transfected with siRNA with empty vector as controls. *P < 0.05 or less vs GC CONT. #*P* < 0.05 or less vs TC CONT. All data were the mean ± s.d. of three independent experiments where all the samples were repeated in thrice.

Since it has been reported that *Bmal1* knockout activated phosphorylation of the PI3K/AKT/mTORC1 pathway (Acosta-Martínez 2011, Guo & Yu 2019), we supposed that it may work as an attractive candidate closely involved in steroidogenic regulation. NfκB, recently proved as a mediator of inducible transcriptional response to circadian signaling, was also reported to have the ability to cross-talk with the PI3K/AKT/mTORC1 (Meng *et al.* 2002, Ghoneum & Said 2019). Based on these evidences, we hypothesized that *Bmal1* might orchestrate luteal steroidogenesis through PI3K/AKT/mTORC1/NfκB pathway. To prove our hypothesis, we examined the phosphorylated proteins of the implicative pathway under the condition of *Bmal1* complete loss in vivo and partially knockdown in vitro. Our results showed that global *Bmal1* deletion *in vivo* resulted in activation of PI3K/AKT/mTORC1 phosphorylation pathway and simultaneously enhanced NfκB phosphorylation at Ser536 in integrated ovary (Fig. 4A).

To strength our findings, phosphorylation of Akt (Thr 308), mTORC1 (Ser 2481), downstream S6K1 at Thr389 together with NfκB at Ser 536 were all detected in both isolated TCs and GCs after *Bmal1*-siRNA transfection. Our results showed that phosphorylation of both pathway PI3K/AKT/mTORC1 and NfκB was activated, which was in accordance with the changes observed in vivo when *Bmal1* was completely deprived. These results confirmed that *Bmal1* impairment leads to over-activation of PI3K/NfκB signaling phosphorylation in luteinzed TCs and GCs (Fig. 4B).

### PI3K/NfκB interacted with negative feedback to BMAL1 on in isolated TCs

To further explore the mechanism under PI3K pathway, isolated TCs was furtherly exposed to LY294002, a selective PI3K inhibitor, post*-Bmal1-*siRNA transfection with LH stimulation. Cellular transcription levels and phosphorylation of NfκB as well as supernatant hormone levels of cultured TCs were examined.

**Figure 5 fig5:**
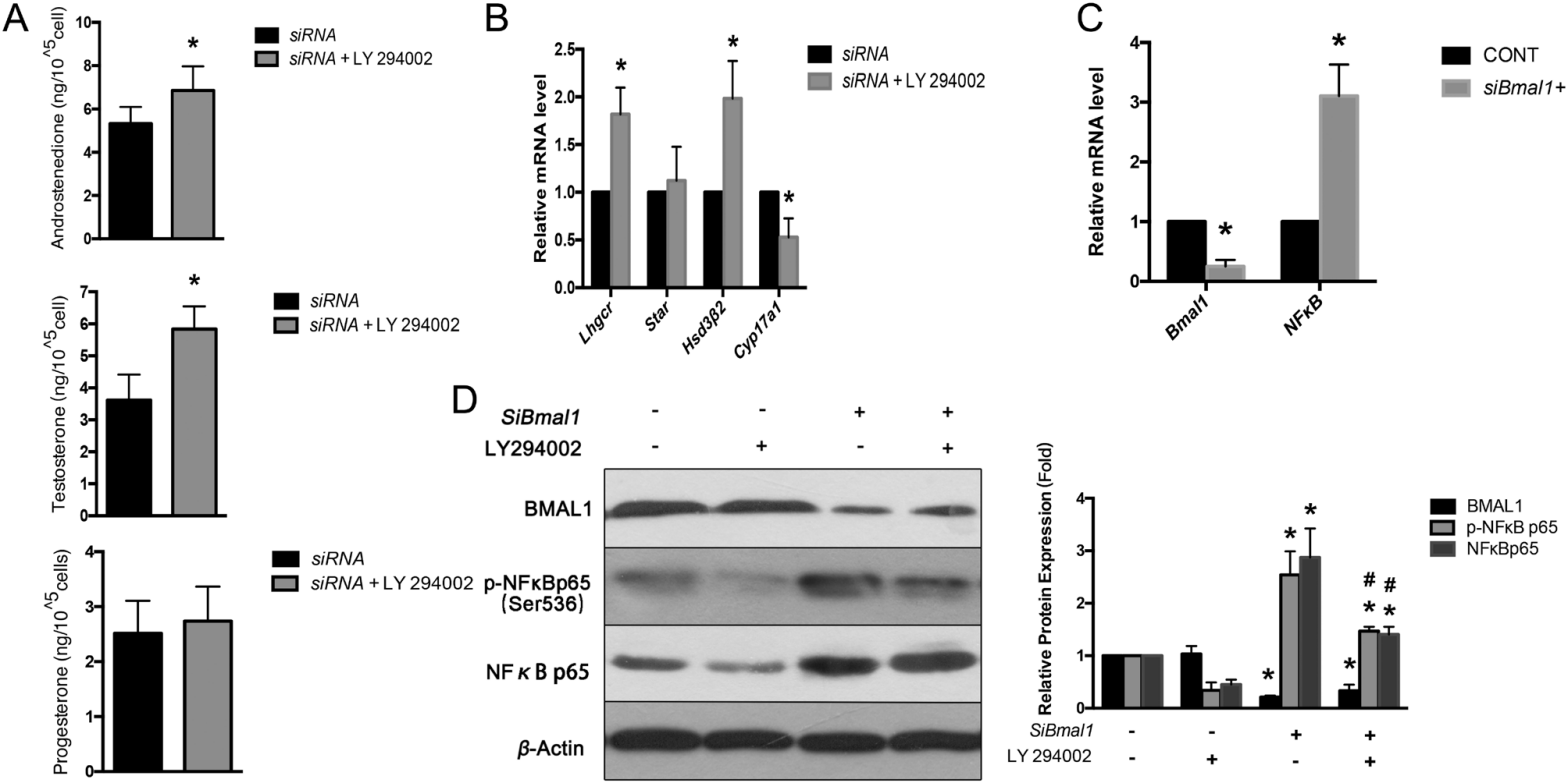
Effects of PI3K specific inhibitor LY294002 on hormone synthesis and underlying NFκB pathway of isolated ovarian theca cells. (A) Androgen and progesterone concentrations in supernatant culture media. (B) mRNA levels of target genes after *Bmal1* siRNA (siRNA) transfection normalized to *Gapdh*. *siRNA*, group of cells transfected with siRNA against *Bmal1* with intact solvent co-cultured as control. mRNA levels of target genes in cells were quantified by qRT-PCR using their specific primers. Data are presented mean ± s.d. of five independent determinations. *P < 0.05 or less vs siRNA. (C) *Bmal1* downregulation enhanced mRNA levels of *NFκB*. (D) *Bmal1* knockdown enhanced total NFκB p65 protein expression and activated phosphorylation of NFκB p65 at Serine-536, which could be suppressed by LY 294002. Left, a typical Western blot. Right, bands were densitometrically qualified and the intensities were normalized to β-actin, relatively to WT control. *P < 0.05 or less vs corresponding protein in *siBmal1*-/LY294002-. #*P* < 0.05 or less vs corresponding protein in *siBmal1*+/ LY294002−. *siBmal1*−, cells transfected with empty vector without *Bmal1* interference. *siBmal1*+, siRNA transfection to interfere *Bmal1*. LY 294002, a PI3K specific inhibitor. LY 294002−, cells treated with mere solvent. LY 294002+, cells co-cultured with solvent dissolved with LY 294002 50 μM for a 2-h incubation at 37°C. Bars represented as mean ± s.d. of three independent determinations.

As shown in Fig. 5A,5 LY294002 intervention significantly rescued AND and T synthesis, together with the elevated trend of P_4_ level, under conditions post*-Bmal1* interference. In accordance with hormonal findings, expression levels of *Hsd3β2* and *Lhcgr* rose with significant differences post-LY294002 intervention while *Star* remained unaffected. Paradoxically, *Cyp17a1* was furtherly reduced (Fig. 5B).

Meanwhile, *Bmal1* exerted a negative feedback on mRNA transcription of NfκB (Fig. 5C). Blockade of PI3K signaling suppressed total protein levels which were enhanced by* Bmal1* knockdown, as well as phosphorylation of NfκB p65 at Ser536 (Fig. 5D).

### Evidences of direct negative interaction between BMAL1 and NfκB

**Figure 6:**
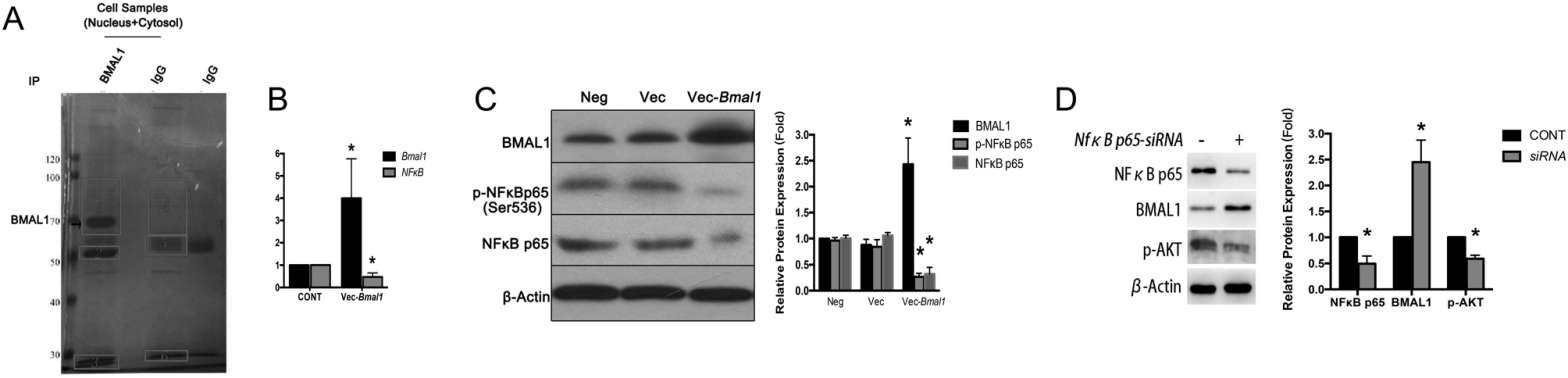
Immunoprecipitations and feedback loops verification of interactions between BMAL1 and NFκB. (A) Immunoprecipitations to characterize proteins that co-precipitated with BMAL1 followed by mass spectrometry in primary-cultured mice theca cells. (B) and (C) Effect of *Bmal1* over-expression on the transcription and protein level of NFκB in mice theca cells. (B) Expression of NFκB quantified by qRT-PCR. (C) Left, a typical Western blot. Right, bands were densitometrically qualified and the intensities shown were normalized to β-actin, relatively to Vec-BMAL1 as control. *P < 0.05 or less vs corresponding protein in Vec. Neg, cells without transfection as negative control. Vec, cells transfected with empty vector as sham control. Vec-*Bmal1*, transfected with vector to overexpress *Bmal1*. Bars represented as mean ± s.d. of three independent determinations. (D) Effects of *NfκB p65* knockdown on BMAL1/PI3K pathway in theca cell. Left, a typical Western blot. Right, bands were densitometrically qualified and the intensities shown were normalized to β-actin, relatively to *NfκB p65-siRNA–* as control. *P < 0.05 or less vs corresponding protein in *NfκB p65-siRNA*–. *NfκB p65-siRNA*– = CONT, cells transfected with empty vector without *NfκB p65* interference. *NfκB p65-siRNA*+ = siRNA, siRNA transfection to interfere NFκB p65 subunit expression. Bars represented as mean ± s.d. of three independent determinations.

To explore clues of direct interaction between BMAL1, mTOC and NfκB, we characterized proteins in nucleus and cytosol of unsynchronized TCs that co-precipitated with BMAL1 through SDS-PAGE (Fig. 6A) followed with mass spectrometry (MS). Primary comparison was conducted between panels from IgG + cell samples and IgG to identify and roll out duplicated proteins, after which the exclusive proteins that co-precipitated with BMAL1 were retrieved from Lane 1. Among candidate proteins, only NfκB p65 (RelA), a subunit of NfκB, was identified. Other retrieved peptides directly interacted with BMAL1 corresponding to annotated mouse proteins were summarized together in Supplementary Excel.

Next, we verified the direct interaction between *Bmal1* and NfκB p65 by *Bmal1* enhancement experiment in TCs. When *Bmal1* was overexpressed, *NfκB* mRNA transcription, corresponding translated proteins and its subunit RelA’ phosphorylation were consistently decreased (Fig. 6B and  C). Combined with our previous results from *Bmal1* knockdown and knockout experiments (Fig. 4A and  B), which showed protein levels of phosphorylated NfκB p65 was upregulated, we proved the negative feedback between *Bmal1* and NfκB p65. It was further verified in *NfκB p65* knockdown experiment, as an obvious increase of BMAL1 protein levels was detected when *NfκB p65* was interfered (Fig. 6D). Additionally, to confirm mediated role of NfκB RelA regarding Bmal1-induced AKT phosphorylation, Western blotting experiments explored the levels of p-AKT. As expected, protein levels of p-AKT was presented to be inhibited in accordance with the changes of NfκB p65 (Fig. 6D). The cellular mechanism regarding *Bmal1* governed NFκB/PI3K pathway in steroidogenesis regulation in TCs was summarized in the schematic diagram (Supplementary Fig. 1, see section on supplementary materials given at the end of this article).

## Discussion

During the past decades, substantial evidences have come to light that ovarian clock orchestrates and synchronizes reproductive physiological events such as follicle aggregation, ovulation and implantation. However, the impact of cellular self-sustaining endogenous circadian rhythms on luteal function is less well studied. The present study showed that deprivation of *Bmal1*, as a molecular-switch of the circadian oscillation, altered luteal phase profile in *Bmal1* null mice. Correspondingly, luteal steroidogenesis was attenuated after *Bmal1* interference, as shown in isolated GCs and TCs, respectively. Molecularly, a novel role of *Bmal1*, which negatively interplayed with PI3K/NfκB in theca cells with respect to regulation of luteal steroidogenesis, was proclaimed.

*Bmal1* exerts its function mainly through central clock, which was proved in *Bmal1* knockout mice. However, previous studies have demonstrated its peripheral role in ovary, for ovarian-specific knockout of *Bmal1* compromised progesterone synthesis, and in vitro study using *Bmal1* downregulation GCs further confirmed the result (Chen *et al.* 2013*b*, Liu *et al.* 2014). However, change of luteal phase profile after *Bmal1* deprivation remained obscure. Here, we showed that global *Bmal1* deprivation could evidently alter mice luteal phase profile, characterized by an advanced peak of P_4_ and minor amplitude of E_2_, indicating possible luteal phase defect. It was noteworthy that an advanced E_2_ level rose at 96 h, a time-point corresponding to the late stage of luteinization followed by a new round of estrus. This metestrus E_2_ priming let us reason that it might be a foreboding of early recruitment of antral follicles for the next round of ovulation, leading to early-onset of ovarian aging detected from female mice with impaired or null *Bmal1* expression (Liu *et al.* 2014). Considering endocrine function of corpus luteum involving not only *Bmal1* but also *Per2* (Shimizu *et al.* 2011, Fahrenkrug *et al.* 2006), another core clock gene which was proved to interact with *Bmal1* in a transcription–translation feedback loop but in a displacement-type manner (Chiou *et al.* 2016), we tested the transcriptional level of *Per2*. Nevertheless, no change of *Per2* expression was found in ovaries of *Bmal1* knockout female mice, which excluded the possible impact of *Per2* in the current study.

To clarify peripheral role of *Bmal1* in the ovary, we used Isolated TCs and GCs, each has independent characteristics with respect to steroidogenesis. Meanwhile, in vitro culture created an environment with no more pulsatile and rhythmic endocrine regulation from hypothalamus and pituitary. Although a bulk of evidences has proved that *Bmal1* was involved in hormone synthesis of ovarian steroid cells, majority focused on GCs (Alvarez *et al.* 2008, Sen & Hoffmann 2020). Given GCs and TCs were born to be with distinctive cellular characteristics and governed underneath diverse endocrine cues, we furtherly explored cell-specific effects raised from *Bmal1* on luteal steroidogenesis. As shown here, evidently impaired AND and T synthesis with a downward trend of P_4_ secretion was detected in TCs. Meanwhile, GCs encountered a significant decreased level of E_2_ secretion, accompanied by a fall of P_4_ in supernatant after partial *Bmal1* loss.

The underlying mechanism of how *Bmal1* modulate the steroidogenesis during luteinization in different type of luteal cells remains unclear. Acting through ovarian clock-controlled genes were proved to be an effective way (Shearman *et al.* 2000), since different clock-controlled elements were detected at the promoters of genes involved in steroidogenesis (Chen *et al.* 2013*b*). In the present study, siRNA-mediated knockdown of *Bmal1* caused downregulation of *Lhcgr* in TCs, which supported existing viewpoint that *Lhcgr* was regulated by circadian clock (Lisa Gilioli *et al.* 2017, Wang *et al.* 2017). Interestingly, exogenous LH stimulation could completely rescue *Lhcgr* expression in isolated *Bmal1*-interferred TCs. In this respect, it might answer why TCKO mice presented with an indistinctive phase shift and abolished rhythm of *Lhcgr* but not a whole loss of its expression, since compensation in TCs might be achieved from endogenous LH secretion in vivo (Mereness *et al.* 2016). However, this impaired-rescued process only worked in TCs other than GCs, as our finding showed that *Lhcgr* of GCs was left impervious after *Bmal1* partially deprivation, consistently with the reported phenotype that GCKO mice persisted with *Lhcgr* diurnal circadian rhythm (Mereness *et al.* 2016). This phenomenon might be attributed to cell-specific characteristics, although we could not exclude the possibility due to incomplete deprivation of *Bmal1* transcriptions, whose residuals were capable enough to sustain considerable sensitivity to LH stimulation. Physiologically, TCs interact directly with ovarian stroma and receiving signals straightforward from peripheral circulation system and the master suprachiasmatic nucleus (SCN) of the hypothalamus (Young & McNeilly 2010). Since GCs locate under the surrounding of TCs, it is reasonable to speculate that intimate cellular interactions worked between GCs and TCs in *in* vivo models may compensate the deficiency of clock function in GCs, leading to the negative result in GCKO mice.

It is worthy to point out that *Bmal1* may exert non-circadian related functions (Liang *et al.* 2013), which is regarded as unique cellular functions distinct or independent from its role in maintaining circadian oscillation (Musiek *et al.* 2013). Although the exact mechanism remains unclear, certain evidences have addressed its non-circadian functions (Yang *et al.* 2016), for example, *Bmal1* knockout mice are characterized with aging properties and metabolic abnormalities, while knockout mice of other clock genes in the core circadian feedback loops, such as *Cry1*/*Cry2* or *Per1*/*Per2* double knockouts, did not present with the same phenotype (Dierickx *et al.* 2018).

As shown in our study, *Bmal1* knockdown activated phosphorylation of PI3K/NfκB pathway, reducing androgen biosynthesis and transcriptional levels of *Hsd3β2* and *Lhcgr*. Blockage of PI3K/NfκB by selective PI3K inhibitor rescued expressions of *Hsd3β2* and *Lhcgr* but exerted no impact on *Star* levels, which sustained the existed perspective that *Star* might be strictly and directly under circadian control rather than PI3k/NfκB signaling. As for *Cyp17a1*, other pathway might be participated under *Bmal1* control, which deserved further exploration (Baburski *et al.* 2016).

It remains elusive how *Bmal1* acted on PI3K pathway. By mass spectrometry in our study, no protein subunit related to mTOC and PI3K was found, indicating that none direct intermediary anchored BMAL1 to mTOC and PI3K, which was in accordance with the results of Wu *et al*. (2019). However, a subunit of NfκB, RelA (also known as NFκB p65) was screened out, which was proposed to anchor as a mediator where BMAL1 exerted regulation to PI3K pathway in a direct manner. The NfκB complex was inclined to be activated during aging process (Salminen *et al.* 2008). Till now, only RelA and RelB from NFκB family have been proved to have direct interactions with BMAL1. Concerning how *Bmal1* interacted with NfκB, to date, very limited evidences were found and none was involved in follicle cells. Here, we proved a negative feedback loop between BMAL1 and NfκB p65, as indicated by converse changes of one to another in the gain and loss experiments, which was consistent with the findings in human aortic endothelial cells by Mengru et al. (Xie *et al.* 2020). However, in mouse embryo fibroblasts (MEFs), the BMAL1-CLOCK complex was detected to directly combine with NFkB p65 subunit, and transcriptionally active form of this specific RelA subunit was proved to be consisted with the dimer overexpression, especially CLOCK dependence (Spengler *et al.* 2012), which elucidated a converse change to our findings. Another subunit RelB of NFκB was found to physically interact with the circadian activator BMAL1 in the presence of CLOCK to repress targeted circadian gene expression (Bellet *et al.* 2012).

Previously findings suggested that NFκB was a downstream of PI3K mediated by AKT (Bai *et al.* 2009), however, latest studies pointed out that there should be a cross-talk between these two cellular signaling (Hussain *et al.* 2012). In our study, by interference the subunit RelA of NFκB and application of PI3K specific inhibitor LY294002, we originally proved a positive cross-talk between NFκB p65 and PI3K/AKT pathway in ovarian TCs mechanistically and functionally, as the luteal steroidogenesis of TCs was correspondingly explored. The precise mechanism on what kind of molecular interaction or modification between Bmal1-induced RelA and PI3K/AKT requires further study.

This study has several limitations. First, we only did *Bmal1* knockdown experiment using siRNA. TCs with *Bmal1* knockout by Crisp*-*cas9 may give us more convincing results, although technically, it is difficult as no stabilized-passaged TC line strain was available. Moreover, if a rescue experiment covering *Bmal1* recover was conducted in vitro, our conclusion might be more conclusive. Lastly, we could not differentiate whether phenotypic changes of TCs were due to disruption of circadian rhythms or the alterations of non-circadian related function of *Bmal1* in the current study, since the rhythm of *Bmal1* in isolated cells was merely weakened but not completely abolished.

To sum up, our study elucidated the significant role of the core circadian gene* Bmal1* in luteal steroidogenesis, mainly interacting negatively but directly with PI3K/NfκB pathway. Our results highlighted the importance of circadian control on reproductive function, which definitely deserves more profound studies in the future.

## Supplementary Material

Fig. 1. Schematic diagram summarizing the Bmal1 regulated NFκB/PI3K pathway in hormone synthesis modulation in mice theca cells.

Supplementary Table

## Declaration of interest

The authors declare that there is no conflict of interest that could be perceived as prejudicing the impartiality of the research reported.

## Funding

This work was supported by National Natural Science Foundation of Chinahttp://dx.doi.org/10.13039/501100001809 (Grant No. 81771588, Youth Program: Grant No. 81801412), Guangdong Basic and Applied Basic Research Foundationhttp://dx.doi.org/10.13039/100007471 (2019A1515010991) and the National Basic Research Program of Chinahttp://dx.doi.org/10.13039/501100012166 (973 Program, Grant No. 2012CB947604).

## Author contribution statement

Xu Yanwen, Wang Yizi and Zhou Canquan designed the research. Wang Yizi, Chen Minghui, Xu Jian, Liu Xinyan and Duan Yuwei performed the specific research. Xu Jian, Liu Xinyan and Duan Yuwei analyzed the data. Xu Yanwen and Wang Yizi wrote the paper. All authors read and approved the final manuscript.
