# Supplementary material for: Core clock gene Bmal1 deprivation impairs steroidogenesis in mice luteinized follicle cells
Source: Reproduction. 2020 Sep 17;160(6):955–67. doi: 10.1530/REP-20-0340 (PMC7707808; doi:10.1530/REP-20-0340)
Supplement: Fig. 1. Schematic diagram summarizing the Bmal1 regulated NFκB/PI3K pathway in hormone synthesis modulation in mice theca cells. [file supplementary_figure_1.pdf]

Supplementary Figure

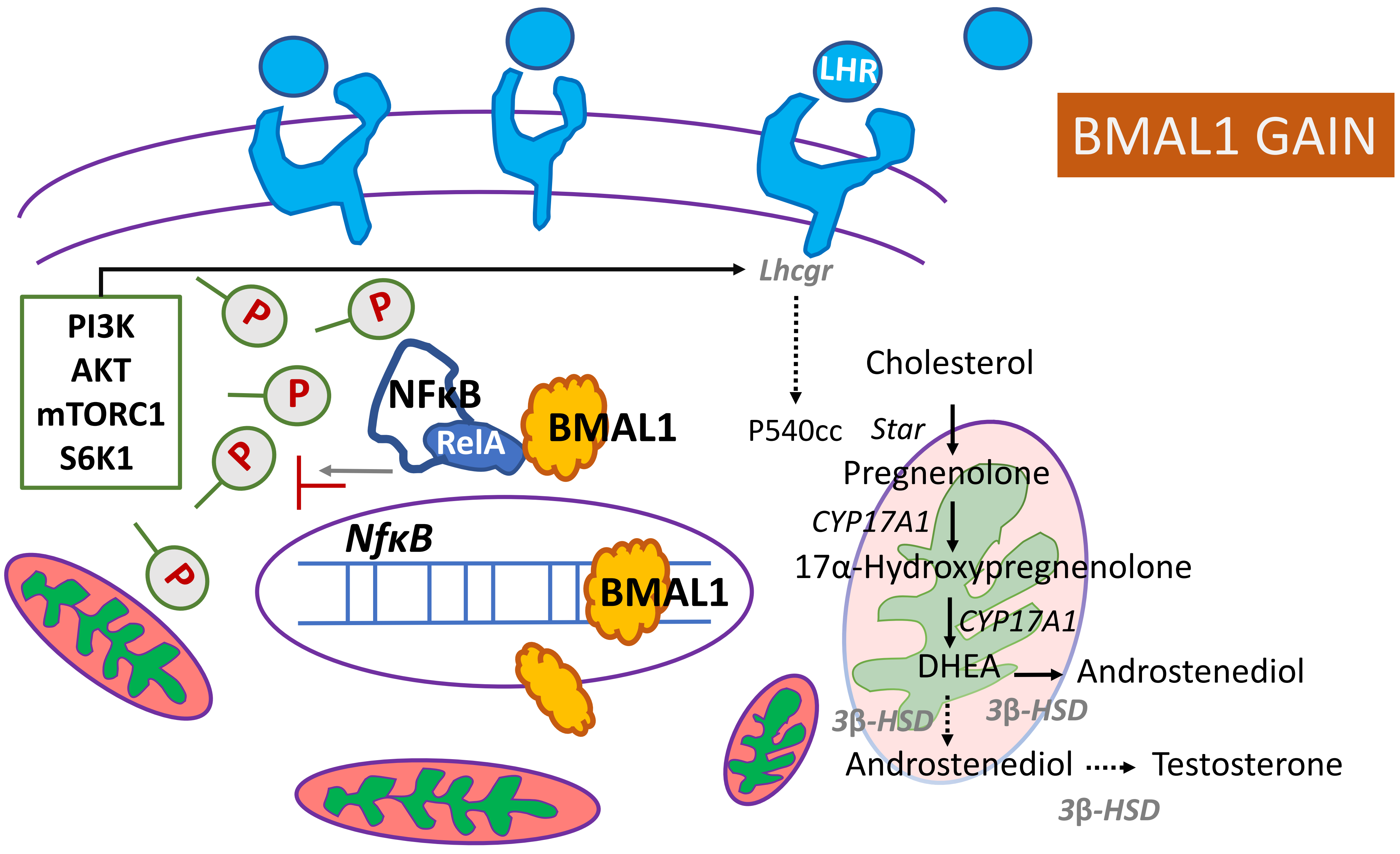

LHR
BMAL1 GAIN
Lhcgr
PI3K
AKT
mTORC1
S6K1
P
P
P
P
P
NFκB
RelA
BMAL1
NfκB
BMAL1
Cholesterol
P540cc
Star
Pregnenolone
CYP17A1
17α-Hydroxypregnenolone
CYP17A1
DHEA
Androstenediol
3β-HSD
3β-HSD
Androstenediol
Testosterone
3β-HSD

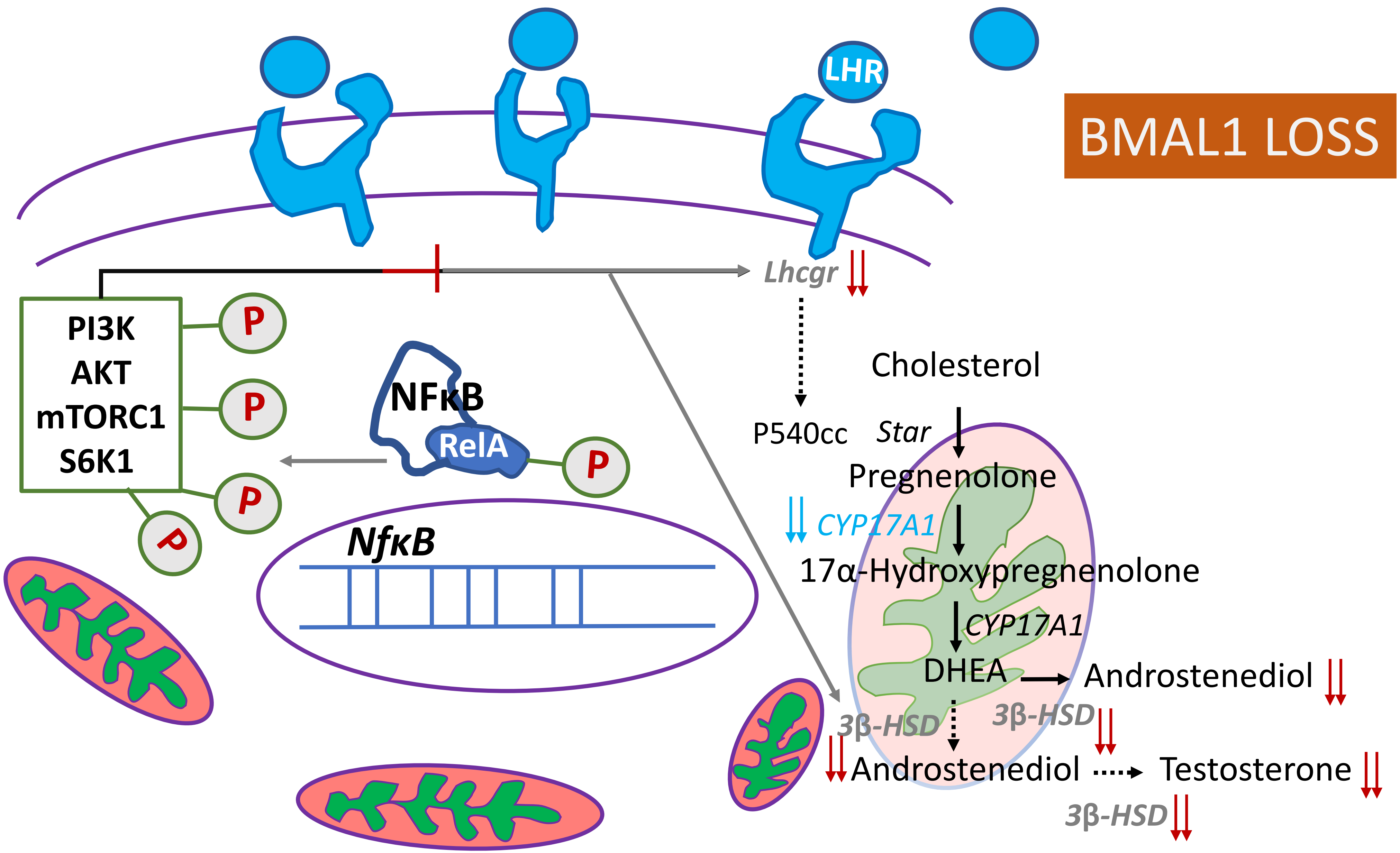

LHR
BMAL1 LOSS
Lhcgr
PI3K
AKT
mTORC1
S6K1
P
P
P
P
NFκB
RelA
P
NfκB
Cholesterol
P540cc
Star
Pregnenolone
CYP17A1
17α-Hydroxypregnenolone
CYP17A1
DHEA
Androstenediol
3β-HSD
3β-HSD
Androstenediol
Testosterone
3β-HSD

Fig. 1. Schematic diagram summarizing the *Bmal1* regulated NFκB/PI3K pathway in hormone synthesis modulation in mice theca cells.
